# Large-scale study for the photocatalytic degradation of paracetamol using Fe_2_O_3_/TiO_2_ nanocomposite catalyst and CPC reactor under natural sunlight radiations

**DOI:** 10.1016/j.mex.2019.11.016

**Published:** 2019-11-18

**Authors:** Omar Fawzi Suleiman Khasawneh, Puganeshwary Palaniandy, Lum Pei Teng

**Affiliations:** School of Civil Engineering, Engineering Campus, Universiti Sains Malaysia, 14300 Nibong Tebal, Pulau Pinang, Malaysia

**Keywords:** Heterogeneous photocatalysis, Nanocomposites, Sol-gel, Hematite, Titanium dioxide, Fe_2_O_3_/TiO_2_ heterogeneous photocatalysis, CPCR, Paracetamol, Emerging contaminants

## Abstract

Heterogeneous photocatalysis is a promising advanced oxidation process for the degradation of emerging contaminants. In this regard, Hematite (α-Fe_2_O_3_) doped TiO_2_ nanocomposite catalyst was synthesized via sol-gel method. The catalyst was prepared in large quantities (225 g) comparatively with other studies and characterized by X-ray diffraction (XRD), Field emission scanning electron microscopy (FESEM), Energy-dispersive X-ray (EDX), and nitrogen gas physisorption studies. The bandgap of the synthesized catalyst was determined using UV–vis diffused reflectance spectroscopy (DRS), and the point of zero charge (PZC) was identified by measuring the zeta potential (*ζ*-*potential*) of the nanoparticles. A large-scale study was conducted using a modified Compound Parabolic Collector Reactor (CPCR) for the degradation of paracetamol under natural sunlight irradiations. The operating parameters including the initial concentration of paracetamol, initial pH of the solution, and catalyst loading were optimized using face-centered central composite design (FCCD) based on response surface method (RSM) to obtain the maximum degradation efficiency of paracetamol.

• The simplified and direct sol-gel method described helps in the synthesis of a novel nanocomposite catalyst (Fe_2_O_3_/TiO_2_) in large quantities while maintaining good characteristics compared to other methods.
• The described treatment method using the modified CPCR will allow the degradation of paracetamol in a more sustainable and green manner.
• Optimizing the operating parameters that have a significant influence on the degradation of paracetamol will contribute towards higher degradation rates.

## Graphical abstract

**Figure:**
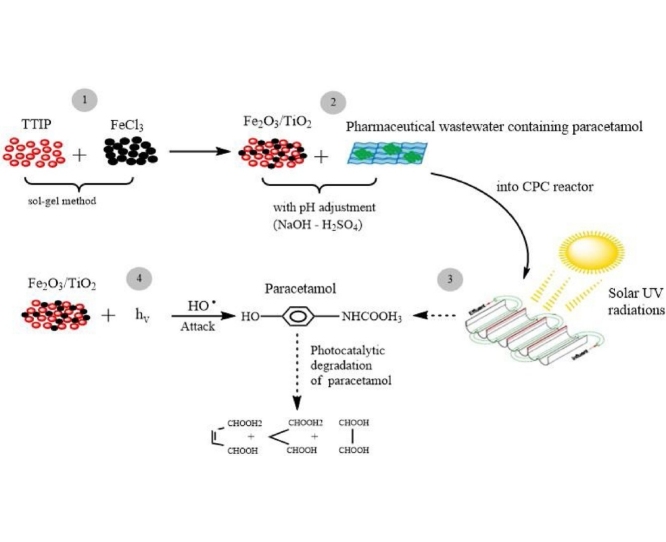

**Specifications Table**

**Table:** 

Subject Area:	Engineering
More specific subject area:	Environmental Engineering
Method name:	Heterogeneous Photocatalysis
Name and reference of original method:	NA
Resource availability:	NA

## Method details

### Phase one: synthesis of Fe_2_O_3_-doped TiO_2_ nanocomposites catalyst

#### Materials

Iron (III) chloride nonahydrate (FeCl_3_) and titanium tetra-isopropoxide (TTIP) 97 % purity were used as precursors for the preparation of the catalyst. Ethanol 95 % was used as an organic solvent, and distilled water was used as a solvent for the FeCl_3_ and to rapid the hydrolysis stage. 37 % hydrochloric acid (HCl) was used to avoid agglomeration of particles. All chemicals were purchased from Sigma-Aldrich Company and used without further treatment.

### Sol-gel process

**Fig. 1:**
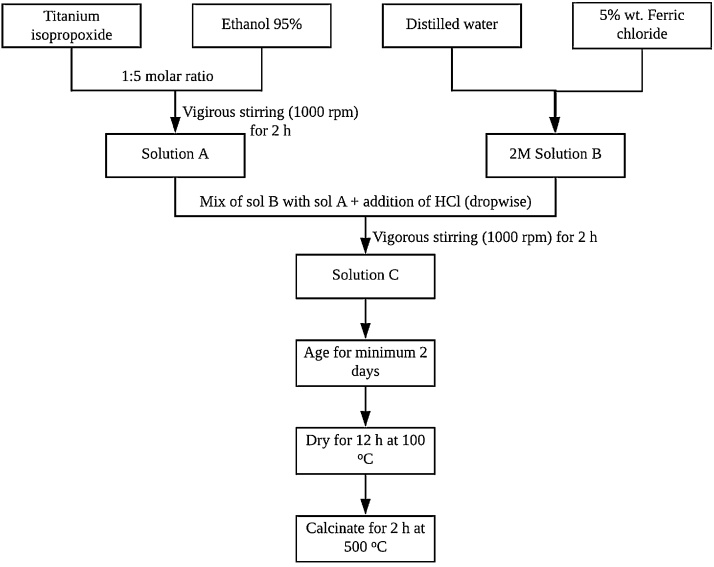
Flow chart for the synthesis of Fe_2_O_3_/TiO_2_ nanocomposite.

α-Fe_2_O_3_ doped TiO_2_ nanoparticles were synthesized via practical and simple sol-gel method. The sol-gel preparation procedures were adopted and modified from several authors [1]. Titanium isopropoxide and ethanol were mixed in the molar ratio of 1:5 and Fe_2_O_3_ dopant content was maintained at 5 wt.%. Fig. 1 illustrates the preparation steps of the Fe_2_O_3_/TiO_2_ nanoparticles.

Here, a predetermined amount of ethanol was poured in a 1-L beaker and then the titanium isopropoxide that corresponds to the molar ratio of 1:5 was added dropwise to the beaker containing Ti/ethanol mixture (Solution A) to avoid the formation of large agglomerates [2]. The mixture (Solution A) was stirred vigorously (1000 rpm) under a magnetic stirrer at room temperature for 2 h. During the addition of TTIP and stirring of solution (A), the formation of white milky solution was noticed. However, suspension color changed during stirring process from white milky to a transparent color or vice versa. This may due to the large amounts of titanium precursor and ethanol used in the synthesizing process of the nanocomposite catalyst. Comparatively, in other experimental work reported by many authors [[3], [4], [5], [6], [7]], the nanocomposite catalyst was produced using a small volume of TTIP and FeCl_3._ It is because in this study, the synthesized nanocomposite catalyst will be used in a compound parabolic collecting reactor (CPCR) as a large-scale study under solar irradiation. FeCl_3_ content of 5 wt.% was dissolved in a precise amount of distilled water (Solution B) then added dropwise to Solution (A) to avoid the agglomeration of particles. The amount of water corresponds to the molar ratio of Ti to H_2_O of 1:0.5. The addition of ferric chloride to Solution (A) resulted in the formation of a thick yellowish solution. The pH of the solution containing Ti and Fe precursors was between 1 and 2. HCl was added in drops after the addition of Solution (B) to avoid agglomeration of the particles and precipitation of TiO_2_ [2,8]. Followed by vigorous stirring for 2 h. The formed sol was poured in glass Pyrex plates and aged for at least 2 days at room temperature. Aging process helps in the oxidation of Fe [7]. After that, the wet gel was dried in the oven for an overnight at 100 °C, then grounded and posteriorly, annealed at 500 °C for 2 h in a muffle furnace. The previous trials conducted for the synthesis of the catalyst as well as the precise amounts of precursors, ethanol, water, hydrochloric acid, and produced catalyst, are shown in Table 1, Table 2 in the attached supplementary material (SP), respectively. Total amount of 225 g of Fe_2_O_3_/TiO_2_ nanoparticles was prepared for the degradation of paracetamol.

**Table 1:** Ranges and levels of the experimental variables.

Experimental variable factors	Label	Unit	Low (−1)	Central (0)	High (+1)
Catalyst loading	Z_1_	g/L	0.5	0.75	1.0
Initial concentration of paracetamol	Z_2_	g/L	0.2	1.1	2
Initial pH of paracetamol solution	Z_3_	–	3	7	11

**Table 2:** Matrix of face central composite design.

Standard order	Run order	Factor 1 Z_1_ (g/L)	Factor 2 Z_2_ (g/L)	Factor 3 Z_3_
3	1	0.5 (−1)	2 (+1)	3 (−1)
6	2	1 (+1)	0.2 (−1)	11 (+1)
12	3	0.75 (0)	2 (+1)	7 (0)
14	4	0.75 (0)	1.1 (0)	11 (+1)
15	5	0.75 (0)	1.1 (0)	7 (0)
2	6	1 (+1)	0.2 (−1)	3 (−1)
8	7	1 (+1)	2 (+1)	11 (+1)
13	8	0.75 (0)	1.1 (0)	3 (−1)
16	9	0.75 (0)	1.1 (0)	7 (0)
10	10	1 (+1)	1.1 (0)	7 (0)
20	11	0.75 (0)	1.1 (0)	7 (0)
19	12	0.75 (0)	1.1 (0)	7 (0)
18	13	0.75 (0)	1.1 (0)	7 (0)
11	14	0.75 (0)	0.2 (−1)	7 (0)
1	15	0.5 (−1)	0.2 (−1)	11 (+1)
5	16	0.5 (−1)	0.2 (−1)	11 (+1)
7	17	0.5 (−1)	2 (+1)	11 (+1)
9	18	0.5 (−1)	1.1 (0)	7 (0)
4	19	1 (+1)	2 (+1)	3 (−1)
17	20	0.75 (0)	1.1 (0)	7 (0)

#### Hints

1 Addition of ferric chloride can cause the solution to become very thick; thus, FeCl_3_ shall be added in drops to avoid the formation of a thick mixture.
2 When a 2-L beaker was used for mixing of the catalyst precursors, the solution was hard to control and for several times, the solution became very thick and barely impossible to stir. This may be attributed to the large amounts of ethanol and titanium precursors used. Therefore, to properly control the synthesis of the catalyst, 1-L beaker was used for the preparation of the catalyst.
3 After the stirring finished, pour the sol in plates made of low-thermal-expansion borosilicate glass (i.e. Pyrex). The sols shall not be placed in plates made of aluminum or metal. As this will induce reactions between the sol and the other material; causing damage to sample.
4 Aging might take more than 2 days; this depends on the amount of the sol prepared. Therefore, it is preferred to pour only little amounts of the sol in the Pyrex plates, to properly control the aging process.
5 Use proper PPE during the preparation of the catalyst as all the materials used are harmful and may cause severe burns or irritation to the eyes and skin.

#### Phase two: characterization

The obtained product (Fe_2_O_3_/TiO_2_ nanocomposites catalyst) was characterized to investigate its structural and optical properties. Generally, the characterization of solid materials involves two processes: structure analysis and property measurements. The structure analysis is accomplished using a variety of microscopic and spectroscopic techniques. Whereas property measurements characterization is more diverse and depends on the individual application. X-ray diffraction (D8 ADVANCE) having Cu Kα (λ = 1.5418 A) radiation as the X-ray source was used to investigate the purity and phase transformation of the prepared nanocomposites. Accelerating voltage of 40 kV and an applied current of 100 mA were subjected to the synthesized nanoparticles during the exposure period to the rays. The XRD patterns of the particles were recorded in the 2θ range from 10° to 80° with a step of 0.02° and integration time of 4 s per step. The phase contents of the prepared Fe_2_O_3_/TiO_2_ nanoparticles were determined by using the following equations [9]:

(1) $$W_{A}=0.886I_{A}/0.886I_{A}+I_{R}$$

(2) $$W_{R}=I_{R}/0.886I_{A}+I_{R}$$

Where w*_A_* and w*_R_* represent the amount of anatase and rutile crystals in the synthesized nanocomposites catalyst, respectively. *I_A_* and *I_R_* present the integrated main peak intensities of anatase and rutile, respectively. Crystalline size of the nanoparticles was estimated using the Scherrer-Debye equation [10]. Field emission scanning electron microscopy (FE-SEM, FEI Quanta FEG 650) was employed to study the evolution of the morphology as well as particle size of the prepared catalyst. For the gold coating, Quorum (Q150 T S) sputter coater was used. The applied accelerating voltage of the SEM was 20 keV [11]. The SEM instrument was equipped with Energy-dispersive X-ray (EDX) to study the percentage composition of iron oxides (Fe^3+^) in the synthesized matrix. The Brunauer – Emmet – Teller (BET) surface area (S_BET_), porosity, pore volume, and Barret – Joyner – Halenda (BJH) pore size and distribution of the nanoparticles were determined based on nitrogen adsorption-desorption isotherms using the Quantachrome AS1Win Analyzer. Prior to the BET surface area measurement, samples were degassed at 150 °C for 2 h under vacuum pressure of (p < 10^−5^ mbar) in the specified port of the analyzer. The degassing temperature has shown to influence the S_BET_, pore volume, and pore size distribution, depending on the material type and properties [12]. Therefore, proper control over the degassing temperature and time is required to ensure the acquisition of accurate results. In order to measure the band gap of the synthesized catalyst, a certain amount of the synthesized nanocomposites catalyst was sonicated in deionized water. UV–vis Diffuse Reflection Spectroscopy (DRS) (Lambda 35) was used to study the optical properties of the nanoparticles by examining the diffuse reflectance spectroscopy (DRS) of the nanocomposite material. Barium sulfate (BaSO_4_) was utilized as a reference and the spectra was noted in the region from 200 nm to 900 nm and the bandgap was determined directly from the UV–vis DRS instrument. The point of zero charge and stability of the synthesized catalyst were identified by measuring the zeta potential of the nanoparticles using the Malvern ZetaSizer. Prior to the zeta potential measurements, a predetermined amount of the nanoparticles (0.015 wt.%) was sonicated in 100 mL of deionized water using the ultrasonic processor (UP200S). Sonication conditions used for the dissolution of the catalyst were fixed at 0.5 cycles and an amplitude corresponding to 55 %.

### Phase three: photocatalytic degradation of paracetamol using the CPC reactor

#### Photocatalytic reactor

**Fig. 2:**
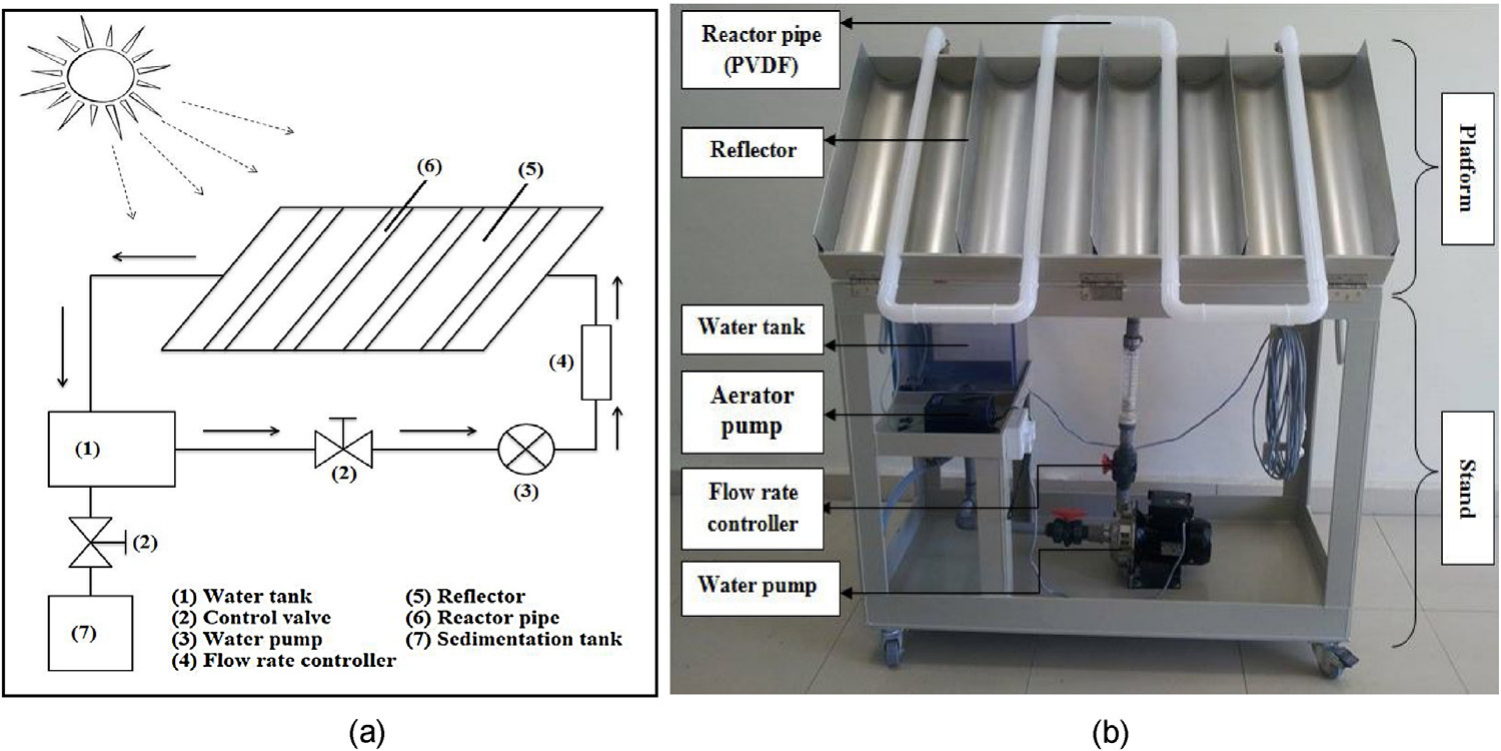
Schematic diagram (a) and a picture of the CPC reactor (b).

The main equipment applied in this study was the Compound Parabolic Collecting Reactor (CPCR). The schematic diagram and a picture of the CPC reactor used in this study are depicted in Fig. 2. The CPCR is considered one of the concentrating solar collector systems [13]. As can be seen from Fig. 2, the main components of CPC reactor are as follows: water tank, control valve, water pump, flow rate controller, reflector and reactor pipes.

The CPC reactor applied in this study was modified from the original CPCR at the Plataforma Solar de Almeria in Spain (PSA), manufactured by Industrial Solar Technology Corporation, Denver, Colorado (USA) [14]. The modification that was done is on the size of the platform. In addition, stone aerators were fixed at the edge of the water tank to improve the circulation of suspension in the water tank and prevent the nanoparticles from settling in the bottom of the water tank. Furthermore, stone aerators act as a bubbler which can increase the concentration of dissolved oxygen in the photocatalysis process, as well as maintaining a homogeneous suspension during the treatment process. The modified CPC reactor composing of two major parts: platform and the stand (Fig. 2b) exhibited a total length of 1200 mm, an overall width of 606 mm, and a height of 762 mm. The platform consisted of eight connected chambers, reflectors, and reactor pipes (see Fig. 2b). The reflectors were made of polished aluminum, allowing it to direct and diffuse the sunlight radiations to the reactor tubes. The reactor tubes were made from polyvinylidene difluoride (PVDF) having a thickness of 2 mm. The PVDF material can endure acids and solvents and allow the passing of the UV light radiations needed for the activation of the photocatalyst.

#### Photocatalytic oxidation procedure

In this study, the synthetic pharmaceutical wastewater was prepared using the commercial *Panadol* (containing 500 mg of paracetamol in each tablet) and deionized water. 3M sodium hydroxide (NaOH) and 3M sulfuric acid (H_2_SO_4_) solutions were used for the adjustment of the pH. All chemicals used were of analytical grade. For each experimental run, 15-L of aqueous synthetic pharmaceutical wastewater containing predetermined amounts of paracetamol were prepared. The pH of the solution containing paracetamol was adjusted to the design value. Followed by the addition of Fe_2_O_3_/TiO_2_ nanocomposites according to the required dosage obtained from the experimental design. The solution was mixed properly to allow a homogeneous suspension. The mixture was then poured into the CPC reactor tank, and stone aerators were placed in the tank to prevent the photocatalyst nanoparticles from precipitating in the uninterrupted part of the water tank and to permit a proper blending between the photocatalyst particles and the synthetic wastewater. Stone aerators also allow more dissolved oxygen in the treatment process through the bubbling process, which can enhance the photooxidation of the paracetamol suspensions by increasing the amount of dissolved oxygen. Additionally, stone aerators help to maintain the homogeneity of the suspensions through the treatment process, which allows more adsorption of the paracetamol particles by the modified photocatalyst.

During the photocatalytic process, the CPC reactor was placed in an open space area with visible and direct sunlight exposure. The platform of the reactor was adjusted according to the local latitude of Penang (5.4 °N) to allow maximum illumination to sunlight radiations. At the same time, the suspension was circulating between the water tank and reactor tubes at a constant flow of 40 L/min by adjusting the horizontal centrifugal pump (MS Series). Low flow rates have shown to provide better degradation of pollutants in the photooxidation process [15].

#### Sample analyzes and photocatalytic performance assessment

The treated synthetic wastewater samples were obtained from the water tank of the CPCR and placed in a container. Samples were taken and filtered using a membrane filter to remove the suspended photocatalyst nanoparticles and to obtain a clear supernatant. The collected supernatant samples were stored in a cold room at 4 °C, and later analyzed for paracetamol concentration using the high-performance liquid chromatography (HPLC) [Shimadzu LC-6A pump, Kromasil 100-5C18 column (4.6 × 250 mm, 5_m)] having a flow rate of 1.0 mL min^−1^ and UV absorbance detection (Waters 481 detector) at 243 nm. The mobile phase was CH_3_OH/H_2_O mixture (30/70, v/v) with an injection volume of 20 μL.

The quantification of pharmaceutical was done by using the peak area integration method (Eq. (3)), and the photocatalytic degradation efficiency was evaluated based on the removal percentage of paracetamol as shown in Eq. (4):

(3) $$\frac{\text{Concentration of the prepared sample}}{\text{Detected peak area of prepared sample}}=\frac{\text{Concentration of external standard}}{\text{Detected peak area of external standard}}$$

(4) $$Removalefficiency\left(\%\right)=\frac{C_{o}-C_{t}}{C_{o}}\times 100\%$$

Where C_o_ and C_t_ represent the initial and final concentrations of paracetamol at time 6 h (g/L), respectively.

#### Experimental design

The optimization of the experimental design study was performed to find the optimum conditions for the degradation of paracetamol in the Fe_2_O_3_/TiO_2_/solar system. Three – variable, three-level Face – Centered Composite Design (FCCD) based on Response Surface Method (RSM) were applied. Design – Expert software, Version 11.0 (Stat Ease, Inc., Mn, USA) was employed for data analysis and response surface optimization. The obtained results were analyzed systematically through the Analysis of Variance (ANOVA) to acquire the relationships between the parameters and response variables. The ANOVA analysis was studied at a 95 % confidence interval in order to analyze the response surface models. The regression models (R^2^) was obtained to measure the fits of the experimental data.

Table 1 shows the treatment conditions applied for this experimental design study. Since the FCCD is non-rotatable, it consisted of three levels noted as: low, central, high, and coded as: −1, 0, +1 (Table 1). The operating factors employed in this study included: (i) catalyst loading, (ii) initial concentration of paracetamol, and (iii) initial pH of the paracetamol solution, labeled as Z_1_, Z_2_, and Z_3_, respectively. The rate of the photocatalytic reaction is significantly influenced by the catalyst concentration. It has been reported that optimum catalyst loading increases the number of active radicals and active sites, thus, leading to enhanced degradation process. However, high catalyst concentrations can decrease the degradation rate due to the increased turbidity, which can inhibit light penetration into the solution matrix needed for the activation of the catalyst surface [3]. In addition, at high pollutant concentration, more pollutant molecules are adsorbed on the surface of the catalyst, and these adsorbed contaminants can restrict photocatalysis by absorbing light before it reaches the surface of the catalyst, resulting in reduced number of active sites and low hydroxyl radicals generation, which leads to poor photocatalytic efficiency [16,17]. Moreover, the initial pH is considered a significant parameter in the photocatalysis process. This is because the pH can influence the adsorption capacity of the pollutant on surface of the catalyst [18]. Other parameters that are likely influence the photocatalytic activity including: (iv) sunlight exposure duration (6 h) [19] and (v) Fe_2_O_3_ dopant content (5 wt.%) [7] were fixed to the most suitable conditions. The operating factors were selected based on their significant influence on the degradation of paracetamol. Although these factors have been studied by several researchers [[19], [20], [21]], however, their results were relatively different. This may be attributed to the model pollutant, type of catalyst, UV source, and sample size employed in their studies. A total number of 20 experimental runs were performed according to the face-centered central composite design and calculated using Eq. (5) which consists of 6 replicate runs at the center, 6 factorial runs, and 8 axial runs. The α-value for this design study was fixed at 1 (face-centered). The experimental response (Y) for this experiment was defined by the degradation efficiency of paracetamol and calculated using Eq. (4). Table 2 shows the matrix of the FCCD experimental design variables utilized in this study to acquire the optimum conditions required for the removal of paracetamol.

(5) $$\text{N}=2^{n}+2n+N_{c}=2^{3}+2\times 3+6=20$$

Where *N* represent the total number of the experimental runs and *n* presents the number of variables.

#### Control study

Control studies were carried out to investigate the UV light photolytic effect on the degradation of paracetamol, adsorption of paracetamol particles on the surface of the prepared photocatalyst (Fe_2_O_3_/TiO_2_), and to study the effect of hydrolysis on the degradation of paracetamol in the absence of photocatalyst and UV light source. Control studies are important to perform to prove that the degradation of paracetamol is due to the photocatalytic activity of Fe_2_O_3_/TiO_2_ nanoparticles.

Three control studies were performed during this research. First control study included the synthetic pharmaceutical wastewater in the presence of UV radiations and in the absence of catalyst Fe_2_O_3_/TiO_2_ (*photolysis*). Second control study involved only Fe_2_O_3_/TiO_2_ nanoparticles in the absence of UV radiations (*adsorption*). Third control study was conducted in the absence of both Fe_2_O_3_/TiO_2_ catalyst and UV radiations (*hydrolysis*). Control studies were conducted in a 1-L beaker that was placed on a magnetic stirrer at room temperature to maintain the homogeneity of the suspensions. The CPC reactor was not used during the control study to avoid any possible engagement between the Fe_2_O_3_/TiO_2_ nanoparticles and paracetamol suspension, which could generate false results. The Fe_2_O_3_/TiO_2_ composites under solar radiations were also examined under the same conditions and results were compared to understand more about the nature of the degradation process of paracetamol.

## Declaration of Competing Interest

The authors declare that they have no known competing financial interests or personal relationships that could have appeared to influence the work reported in this paper.
