# Supplementary material for: Large-scale study for the photocatalytic degradation of paracetamol using Fe2O3/TiO2 nanocomposite catalyst and CPC reactor under natural sunlight radiations
Source: MethodsX. 2019 Nov 18;6:2735–43. doi: 10.1016/j.mex.2019.11.016 (PMC6879991; doi:10.1016/j.mex.2019.11.016)
Supplement: Supplementary file 1 [file mmc1.docx]

# **Supplementary material**

Table 1 Trial tests for synthesis of nanocomposite catalyst samples

| Catalyst | Molar ratio of TTIP to EtOH | Amount of Fe_2_O_3_ (%wt.) | pH of solution | Molar ratio of TTIP to H_2_O | Comments |
| --- | --- | --- | --- | --- | --- |
| A | 0.5M | 5 wt.% | Adjusted to 9 using 6M NaOH | 1 : 0.5 | Not practical to use; as large amounts of NaOH were added to adjust the pH to the required value.  The addition of NaOH rendered the sol very thick which prohibited the control of the sols during stirring. |
| B | 1 : 10 | 5 wt.% | Original pH ~1.5 | 1 : 0.5 | Nanocomposite catalyst prepared in normal conditions (not acidic nor basic) exhibited small surface area and large particle size distribution. |
| C | 0.5M | 5 wt.% | Original pH ~1.5 | 1 : 1 | Nanocomposite catalyst prepared in normal conditions (not acidic nor basic) exhibited small surface area and large particle size distribution. |
| D | 1 : 4 | 5 wt.% | Adjusted to 3 using 3M NaOH | 1 : 0.5 | Similar to sample (A), large amounts of NaOH were added to adjust the pH to 3 and the formation of a thick solution was also observed. |
| E * (selected catalyst) | 1 : 5 | 5 wt.% | Acidic | 1 : 0.5 | For this sample, HCL was added in drops (3 drops for each 10 mL of TTIP added). The addition of HCL prevents the agglomeration of catalyst nanoparticles.  Formation of a relatively thick solution was observed during the addition of HCL.  Samples prepared under acidic conditions exhibited the largest surface area and the smallest particle size. |

**Amount of TTIP was calculated using molarity (M) equation:**

$M=\frac{moles(TTIP)}{mL\text{ }solution}$ (1)

Where,

$Moles=\frac{weight\text{ }of\text{ }TTIP}{molecular\text{ }weight\text{ }of\text{ }TTIP}$ (2)

Amount of FeCl_3_ to be added to make a 5 wt.% was calculated according to the following equation:

$wt.\%=\frac{solvent}{solute}$ (3a)

$wt.\%=\frac{weight\text{ }of\text{ }FeCl_{3}\text{ }(g)}{(density\text{ }TTIP\times mL)\text{ }+\text{ }(density\text{ }EtOH\times mL)\text{ }+\text{ }water\text{ }(mL)}$ (3b)

The amount of FeCl_3_ obtained from Equation (3b) shall be divided by 2, according to the balanced chemical reaction shown in Equation (4):

$2FeCl_{3}+\text{ }3H_{2}O\to Fe_{2}O_{3}+\text{ }6HCl$ (4)

Where the molar ratio of FeCl_3_ to Fe_2_O_3_ is 2:1.

* Sample (E): for each 1-L beaker, the following amounts of chemicals were added and mixed (see Table 2). A total amount of 30 grams were produced for each 150 mL of titanium isopropoxide and according to the mentioned conditions. This amount was validated each time the catalyst was prepared. As prior to the preparation of large amounts, a sample test trials were performed using 10 mL of titanium isopropoxide and with the same conditions. A total amount between (2 – 2.2 g) was produced for each trial.

Table 2 amounts of precursors and chemicals used for the preparation of the catalyst (sample E)

| Chemical | Amount |
| --- | --- |
| Titanium isopropoxide (TTIP) | 150 (mL) |
| Ferric chloride (FeCl3) | 5 wt.% = 20.2 (g) |
| Ethanol 95% | 750 (mL) |
| Distilled water | 75 (mL) |
| Hydrochloric acid (HCL) | 3 drops for each 10 (mL) of TTIP |
